# Comparative RNA-Sequence Transcriptome Analysis of Phenolic Acid Metabolism in* Salvia miltiorrhiza*, a Traditional Chinese Medicine Model Plant

**DOI:** 10.1155/2017/9364594

**Published:** 2017-01-17

**Authors:** Zhenqiao Song, Linlin Guo, Tian Liu, Caicai Lin, Jianhua Wang, Xingfeng Li

**Affiliations:** ^1^Agronomy College, Shandong Agricultural University, Tai'an, Shandong 271028, China; ^2^State Key Laboratory of Crop Biology, Agronomy College, Shandong Agricultural University, Tai'an, Shandong 271018, China

## Abstract

*Salvia miltiorrhiza *Bunge is an important traditional Chinese medicine (TCM). In this study, two* S. miltiorrhiza* genotypes (BH18 and ZH23) with different phenolic acid concentrations were used for de novo RNA sequencing (RNA-seq). A total of 170,787 transcripts and 56,216 unigenes were obtained. There were 670 differentially expressed genes (DEGs) identified between BH18 and ZH23, 250 of which were upregulated in ZH23, with genes involved in the phenylpropanoid biosynthesis pathway being the most upregulated genes. Nine genes involved in the lignin biosynthesis pathway were upregulated in BH18 and thus result in higher lignin content in BH18. However, expression profiles of most genes involved in the core common upstream phenylpropanoid biosynthesis pathway were higher in ZH23 than that in BH18. These results indicated that genes involved in the core common upstream phenylpropanoid biosynthesis pathway might play an important role in downstream secondary metabolism and demonstrated that lignin biosynthesis was a putative partially competing pathway with phenolic acid biosynthesis. The results of this study expanded our understanding of the regulation of phenolic acid biosynthesis in* S. miltiorrhiza.*

## 1. Introduction

*Salvia miltiorrhiza *Bunge is a widely used traditional Chinese medicine (TCM) with multiple clinical and pharmacological effects associated with cardiovascular, cerebrovascular, and hyperlipidemial diseases [1]. Meanwhile* S. miltiorrhiza* is also an important model plant for TCM studies due to its short life cycle, small genome size, undemanding growth requirements, and significant medicinal value [2]. The major bioactive components of* S. miltiorrhiza *are diterpenoids, flavonoids, sterols, and water-soluble phenolic acids [3].

The water-soluble phenolic acids in* S. miltiorrhiza* primarily contain salvianolic acid B (Sal B), rosmarinic acid (RA), caffeic acid, Danshensu, 4-coumaric acid, and t-cinnamic acid. Sal B is prevalent in water-soluble extracts and exhibits abundant cardioprotective effects. Thus, Sal B is used as a chemical marker of* S. miltiorrhiza* roots and acts as a phytomedicine. The current Chinese Pharmacopoeia requires the Sal B content in dry* S. miltiorrhiza* roots higher than 3.0% (CPA, 2015) [4]. Preliminary investigations have shown that Sal B concentration varies greatly in different genotypes [5–8]. However, the reason for these differences has not yet been investigated to date.

Sal B is presumed to be synthesized from RA [9], and the biosynthetic pathway of RA has been characterized in plants (Figure S1 in the Supporting Information in Supplementary Material available online at https://doi.org/10.1155/2017/9364594). RA biosynthesis begins with the aromatic amino acids, L-phenylalanine and L-tyrosine, which are separately converted to the intermediate precursors 4-coumaroyl-CoA and 4-hydroxyphenyllactic acid through the tyrosine-derived pathway and the phenylpropanoid pathway, respectively. These two intermediate precursors are then covalently coupled by several biological reactions to generate RA [10]. A number of key enzymes involved in producing the RAs in* S. miltiorrhiza* have been identified [11–13], while the detailed pathway from RA to Sal B has not yet been characterized.

In recent years, several kinds of transcriptome sequencing platforms such as Sanger sequencing, 454 pyrosequencing, and Solexa sequencing have been used for discovering related genes involved in secondary metabolism pathways of* S. miltiorrhiza*. However, previous studies were mainly focused on tanshinones biosynthesis and its regulation [14–18]; few reports were focused on the phenolic acid biosynthesis [14, 16, 19]. Furthermore, those previous RNA-seq studies were focused only on the development stages, tissue types, and hairy root cultures of one specific genotype; studies focused on different genotypes with diverse concentrations of bioactive components have not been conducted.

Compared with forward sequencing methods, Illumina RNA-seq technology has been proved as a more effective approach for transcriptome study, with higher read depth and prediction accuracy capabilities [20]. However, it is seldom used in* S. miltiorrhiza* transcriptome study.

In this study, two different lines (ZH23 and BH18) were bred through individual selection from the natural germplasm of* S. miltiorrhiza*; the Sal B content in ZH23 roots was significantly higher than that in BH18. In the present study, Illumina RNA-seq technology is used to investigate the transcriptome profile of these two lines, with the goal of unraveling the differentially expressed genes (DEGs) involved in phenolic acid biosynthesis pathway. The results of this study would expand our understanding of the regulation of phenolic acid biosynthesis in* S. miltiorrhiza*.

## 2. Materials and Methods

### 2.1. Plant Materials

**Figure 1:**
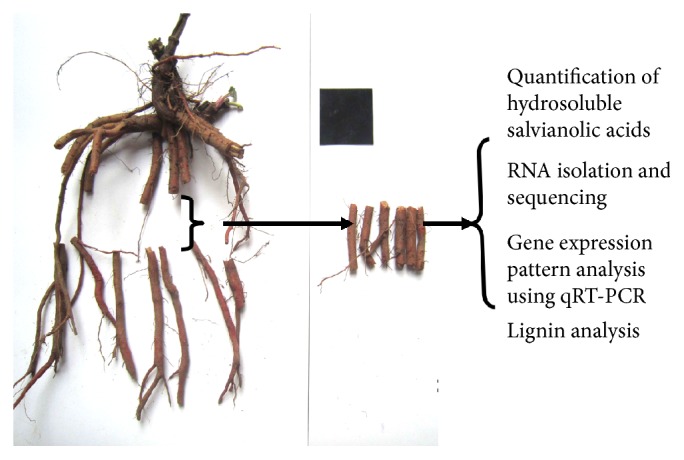
The root segments of* S. miltiorrhiza* for further analysis.

Two different* S. miltiorrhiza* lines, BH18 and ZH23, were used in this study. Each line is asexually propagated through root cutting. Thirty individuals of each genotype were planted in 5 m^2^ plots and were grown under identical conditions at the Botanical Garden in the Agronomy College of Shandong Agricultural University. Mid-segments roots with a length of 5 cm were collected from 9 randomly selected individual plants for each line at the harvest stage (October 25th, 2014) and divided into four parts (Figure 1): The first part was mixed with equivalent fresh weight for RNA sequencing. Another part was used for RNA isolation and real-time PCR analysis. All the above samples were frozen immediately in liquid nitrogen and stored at −80°C until further processing. The third part was used to determine phenolic acid content, and the remaining part was used for lignin analysis.

### 2.2. Extraction, Analysis, and Quantification of Hydrosoluble Salvianolic Acids Using High-Performance Liquid Chromatography (HPLC)

The collected roots of both genotypes were dried at 30°C to constant weights and then ground into powder and 100 mg powder of each line was extracted with 50 mL methanol in an ultrasonic bath for 1 hr. The supernatant was analyzed by HPLC after filtration through a 0.22 *μ*m membrane. Each experiment was run in triplicate. The HPLC analyses of the phenolic acids were performed as described by the CPA (2015) [4].

### 2.3. RNA Isolation and Quality Verification

To eliminate the variation between individual plants, the RNA were isolated from mixed root segments of three individual plants and three RNA samples were extracted for each line. For RNA extraction, the collected roots were homogenized in liquid nitrogen first, and then the total RNA was extracted using TRIzol@ Reagent (Invitrogen, San Diego, USA) and treated with DNaseI (Invitrogen). The quantity and quality of the extracted RNA were verified by gel electrophoresis and a NanoPhotometer® spectrophotometer (IMPLEN, CA, USA).

### 2.4. cDNA Library Construction and Sequencing

Equivalent quantities of total RNA from three samples were mixed to prepare the pooled RNA samples for RNA-seq. A total quantity of 30 *μ*g of mixed RNA was confirmed for RIN values above 8.0 and was used as input material for the construction of RNA-seq library. The library was generated using the Illumina TruSeq™ RNA Sample Preparation Kit (Illumina, San Diego, USA) following the manufacturer's recommendations. The clustering of the index-coded samples was performed on a cBot Cluster Generation System using the TruSeq PE Cluster Kit v3-cBot-HS (Illumina) according to the manufacturer's instructions. After cluster generation, the library preparations were sequenced on an Illumina Hiseq 2000 platform, and 100 bp paired-end reads were generated.

### 2.5. Data Processing, Assembly, and Annotation

Clean data (clean reads) were obtained by removing the reads containing adapters, the reads containing poly-N, and low-quality reads, from the raw data. The Q20, Q30, GC-content, and sequence duplication levels of the clean data were calculated. All of the downstream analyses were based on high-quality clean data. The left read files (read1 files) from all of the libraries/samples were pooled into a single left.fq file, and the right read files (read2 files) were pooled into a single right.fq file. The transcriptome assembly was accomplished based on the left.fq and right.fq files, using Trinity [21] with min_kmer_cov set to 2 and all of the other parameters set to default.

All of the assembled unigenes of the two lines were searched against the NCBI Nr database to identify their putative mRNA functions using the BLAST algorithm [22] with an *E*-value cut-off of 10^−5^. Additionally, Gene Ontology (GO) terms were extracted from the strongest hits obtained from BLASTx against the Nr database using the Blast2GO program [23]. The BLAST algorithm was also used to align unique sequences to Nt, Pfam, KOG, Swiss-Prot (manually annotated and reviewed protein sequence database), COG (Clusters of Orthologous Groups of proteins), and KO (KEGG Ortholog database) [24] (with an *E*-value cut-off of 10^−5^) to predict possible functional classifications and molecular pathways.

### 2.6. Gene Expression Pattern Analysis

Gene expression levels were estimated by mapping clean reads to the Trinity transcripts assembly using RSEM [25] for each sample. The abundance of all of the genes was normalized and calculated using uniquely mapped reads with the RPKM method [26]. A differential expression analysis of the two samples was performed by modeling count data with negative binomial distributions, as described in the DEGseq method [27]. *P* values were adjusted using *Q* values [27], and [*q* value < 0.005  and  |log_2_⁡(fold  change)| > 1] was set as the threshold for a significant differential expression level. The identified differentially expressed genes (DEGs) were used for the GO and KO enrichment analyses. The GO enrichment analyses were performed using GOseq [28], based on the Wallenius noncentral hypergeometric distribution, to map all of the DEGs to terms in the GO database (*P* value ≤ 0.05) to identify significantly enriched GO terms in the DEGs. The KEGG pathway enrichment analysis of the DEGs was completed using KOBAS [29].

### 2.7. Real-Time Quantitative RT-PCR (qRT-PCR) Assay

Gene expression levels were determined via qRT-PCR [30]. A housekeeping gene,* Ubiquitin*, was used as a control to account for the variation in the cDNA template levels. The target and reference cDNAs were amplified with identical reaction mixtures in a single iCycler iQ5 run. Gene-specific primers for quantitative PCR are listed in Table S1 in the Supporting Information.

### 2.8. Lignin Analysis

The main roots from the harvest stage were used for analyzing the lignin contents of both lines. Free-hand sections were immediately immersed in 5% phloroglucinol (dissolved in 100% ethanol) for 2 min and then incubated in concentrated HCl. The samples were then photographed within 30 min [31]. The Klason lignins were then extracted and measured according to the protocol of Zhang et al. [32].

## 3. Results

### 3.1. The Content of Phenolic Acid Component in ZH23 and BH18

**Figure 2:**
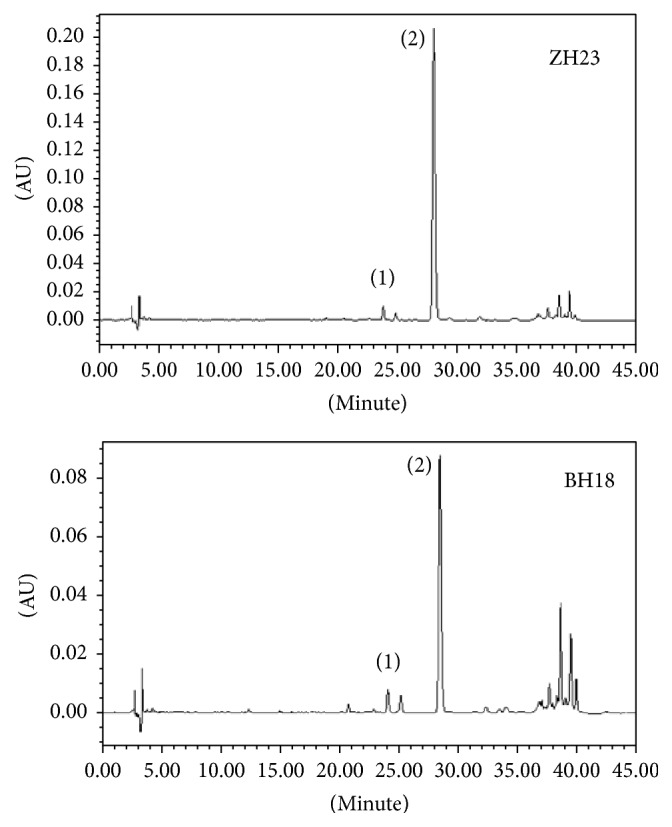
Elution profile of ZH23 and BH18 on the HPLC-UV chromatograms. The retention times for RA (1) and tanshinone Sal B (2) were 23.901 min and 28.303 min, respectively.

The phenolic acid concentrations of ZH23 and BH18 roots at the harvest stage were determined via HPLC (Figure 2). Seven common peaks of phenolic acid components were obtained in both lines, in which two peaks were identified as Sal B and RA according to the consistency of retention time and spectra of the peaks (Figure 2). The total peaks area were considered to be the total salvianolic acid. Results showed that the content of Sal B, RA, and the total phenolic acid in ZH23 were higher than those of BH18 (Table S2). The ratio of Sal B in total phenolic acid was also higher in ZH23 (about 75%) than that in BH18 (60%). In both lines, Sal B was found to be the main compounds.

### 3.2. RNA-seq and De Novo Assembly of ZH23 and BH18

Mixed root samples at the harvest stage of ZH23 and BH18 were used for RNA-seq analysis. After quality control and the removal of low-quality reads, a total of 81,665,757 and 61,924,629 clean reads were attained from BH18 and ZH23 cDNA libraries. More than 90% of the clean reads had a read quality of Q30 (sequencing error rate 0.1%) or higher. Due to the absence of genomic reference sequences, de novo assembly was applied while the clean reads from both lines were pooled together, resulting in 170,787 transcripts and 56,216 unigenes with N50 lengths of 2,026 and 1,577, respectively.

The unigene annotations were performed by BLAST searches (*E*-value ≤ 10^−5^). A total of 25,921 unigenes (46.11% of the unigenes) were annotated with significant BLAST results in the Nr database; 20,128 unigenes (36.59% of the unigenes) were annotated in the Swiss-Prot database; 20,574 unigenes were annotated in Pfam, and 22,424 unigenes were annotated in the GO database. In total, 28,707 unigenes were annotated in at least one database (Table 1). To further identify the active biological pathways in* S. miltiorrhiza*, 9,162 unigenes annotated by BLAST analysis against the KAAS (KEGG Automatic Annotation Server) were mapped to 250 reference canonical pathways. These canonical pathways were classified into five main categories: “cellular processes,” “environmental information processing,” “genetic information processing,” “metabolism,” and “organismal systems” (Figure S2 in the Supporting Information). The pathways with the highest representation were “translation” (937 unigenes, 10.23%) and “carbohydrate metabolism” (832 unigenes, 9.08%). These annotations and classifications provided a resource for investigating specific pathways in* S. miltiorrhiza*, such as the RA biosynthetic pathway. The biosynthetic pathway leading to Sal B and RA is thought to entail both phenylpropanoid and tyrosine-derived pathways. Totally, 96 unigenes were clustered into “phenylpropanoid biosynthesis”; 52 and 51 unigenes were classified into the “tyrosine metabolism” and “phenylalanine, tyrosine, and tryptophan biosynthesis” subpathways, respectively.

### 3.3. RPKM Density Distribution of Transcript Profiling

The two genotypes showed similar RPKM density distributions, which suggested that their transcription profiles were similar (Figure S3 in the Supporting Information). The top two most abundant transcripts in the roots, which accumulated at 32,104 RPKM, were the* S. miltiorrhiza* SMLII mRNA, which is consistent with previous transcriptome data using 454 GS FLX from roots grown for 2 years [15].

### 3.4. DEGs of the Two Genotypes

**Figure 3:**
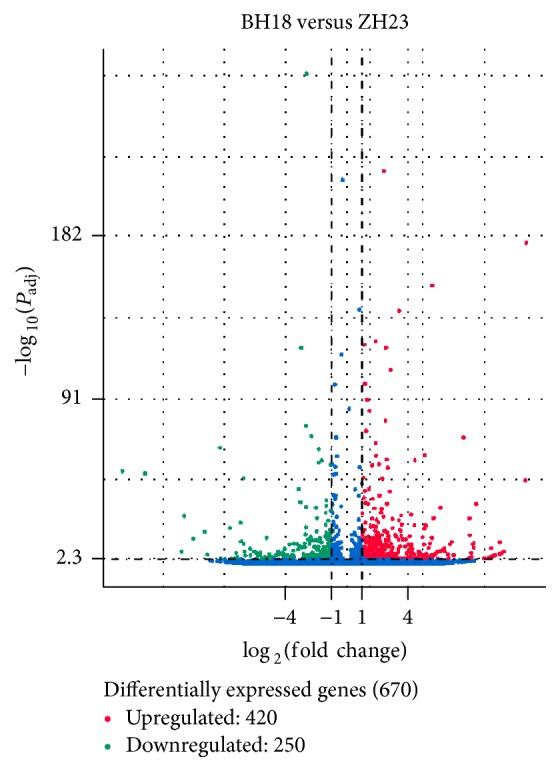
Comparison of expression patterns of differential unigenes identified between BH18 and ZH23. The red dots represent upregulated DEGs, the green dots represent downregulated DEGs, and the blue dots represent non-DEGs. In total, 670 unigenes were identified as differentially expressed genes (*Q* value < 0.005  and  log_2_(fold change) > 1) between BH18 and ZH23.

On the basis of the applied threshold [*q*-value < 0.001 and log_2_⁡(fold change) > 1], 670 genes (1.19% of the total genes) were identified as significant differentially expressed genes (DEGs) between these two lines, which comprised 250 upregulated genes (accounting for 37.32% of the significant DEGs) and 420 downregulated genes (62.68%) in ZH23, respectively (Figure 3).

**Figure 4:**
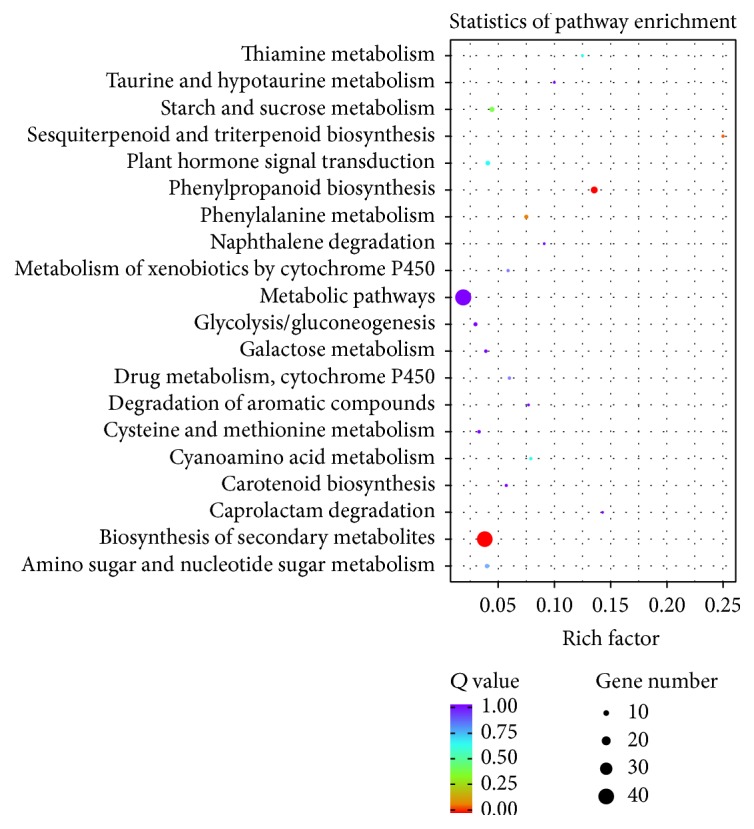
Scatterplot of differentially expressed genes enriched in the top 20 KEGG pathways. Rich factor represents the ratio of the number of DEGs and the number of all unigenes in the pathway; *Q* value represents corrected *P* value.

To investigate the biochemical pathways of the DEGs, we mapped the DEGs in terms in the KEGG database and compared these results with the complete transcriptome background.  Figure 4 shows a scatterplot of the DEGs that were enriched in the top 20 KEGG pathways. Of these, two pathways, “phenylpropanoid biosynthesis” (Corrected *P* value 0.00000117) and “biosynthesis of secondary metabolites” (Corrected *P* value 0.000059), were the most significantly enriched pathways.

### 3.5. DEGs Involved in the Phenolic Biosynthesis Pathway

**Figure 5:**
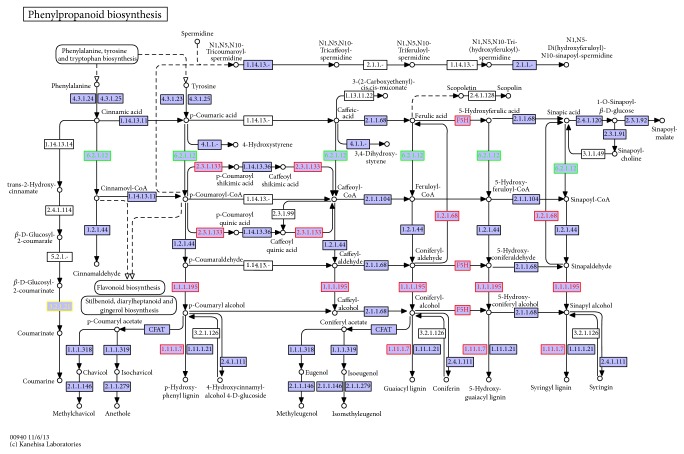
Representation of genes related to phenolic acid biosynthesis. Note: the red frames and red letters or numbers represent upregulated DEGs in BH18; the green frames and green letters or numbers represent downregulated DEGs in BH18; the purple frames represent non-DEGs.

The biosynthetic pathway leading to Sal B and RA is thought to entail both phenylpropanoid and tyrosine-derived pathways. In total, 11 DEG unigenes were related to phenylpropanoid biosynthesis or related pathways.  Figure 5 shows a representation of the DEG unigenes related to phenolic acid biosynthesis and the competing pathways.

Among the 11 DEGs, one DEG, comp49431_c1, was upregulated 1.582-fold in ZH23 and was annotated as “4-coumarate:CoA ligase 2 (*Sm4CL2*, EC 6.2.1.12),” which is involved in the core-phenylpropanoid pathway. Enzyme 4CL plays an important role in the general phenylpropanoid pathway by catalyzing a series of aromatic substrates to form their corresponding hydroxycinnamoyl-CoA esters, which are key precursors for the biosynthesis of numerous phenylpropanoid derivatives, including RA, flavonoids, and lignin [33].

The other DEGs were all downregulated in ZH23 and upregulated in BH18, and those genes were associated with the branched lignin biosynthesis pathway. Among them, comp50099_c1, which was annotated as shikimate O-hydroxycinnamoyl transferase (HCT, EC: 2.3.1.133), had an expression level in BH18 that was 2.17-fold higher than ZH23. HCT catalyzes the synthesis of the shikimate and quinate esters of p-coumaric acid [34], which controls the biosynthesis of two major lignin building units, namely, the guaiacyl (G) and syringyl (S) units. Comp51337_c0 (1.1205), which was annotated as “ferulate-5-hydroxylase (F5H, EC: 1.14.-.-),” a cytochrome P450-dependent monooxygenase (P450) of the general phenylpropanoid pathway [35], was upregulated 1.12-fold in BH18. F5H catalyzes the hydroxylation of ferulic acid, coniferaldehyde, and coniferyl alcohol in the pathways leading to sinapic acid and the syringyl unit of lignin [36]. Comp24721_c0, which was annotated as “coniferyl-aldehyde dehydrogenase (REF1, EC: 1.2.1.68),” was upregulated 3.527-fold and catalyzes coniferyl aldehyde to ferulate acid. Two unigenes, comp48837_c0 (2.1147-fold) and comp42506_c0 (1.2278-fold), were annotated as “cinnamyl-alcohol dehydrogenase (CAD, EC: 1.1.1.195)”. CAD specifically catalyzes the reduction of cinnamaldehydes to cinnamyl alcohols during the final step of monolignol biosynthesis. Several homologs of CAD play important roles in the lignification of elongating stems in* Arabidopsis* [37]. In addition, five unigenes were annotated as “peroxidase (POD, EC: 1.11.1.7)” also upregulated in BH18, including comp23935_c0 (3.361-fold), comp49112_c0 (2.2654-fold), comp24685_c0 (4.1104-fold), comp36960_c0 (1.7491-fold), and comp45749_c0 (4.0349-fold). The last major step in lignin synthesis involves monolignol dehydrogenation and polymerization, and different classes of oxidative enzymes are implicated in this step, including class III peroxidase (POD; EC 1.11.1.7), laccase, ascorbate peroxidase, and NADPH oxidase [38].

### 3.6. The Expression Profiles of Genes Related to RA Biosynthesis

**Figure 6:**
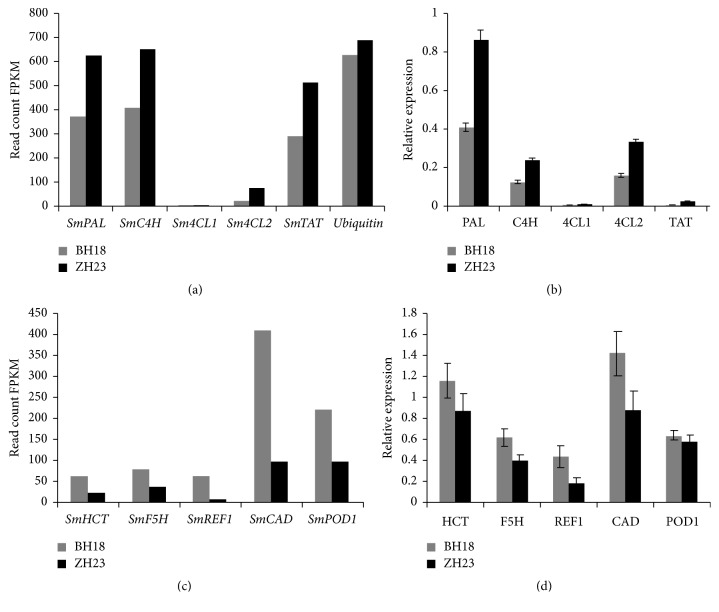
Expression patterns of the genes involved in phenolic acid biosynthesis in BH18 and ZH23 detected by DGE and validated by qPCR. (a) RA biosynthesis genes detected by digital gene expression (DGE). (b) The 5 genes selected from above were confirmed by qPCR. (c) Lignin biosynthesis genes detected by digital gene expression (DGE). (d) The 5 genes related with lignin biosynthesis genes confirmed by qPCR. The results represent the means ± standard deviations of experiments performed in triplicate.

The gene expression patterns of* SmPAL*,* SmC4H*,* Sm4CL1*,* Sm4CL2*,* SmTAT*, and* SmHPPR*, which are reported to be associated with RA biosynthesis, were validated by qRT-PCR.* Ubiquitin* was used as an internal reference. The transcription levels of these genes were low in both lines compared with the internal reference gene (Figures 6(a)-6(b)). The expression levels of* SmPAL*,* SmC4H*,* Sm4CL1*,* Sm4CL2*,* SmTAT*, and* SmHPPR* in ZH23 were all significantly higher than that in BH18, which were generally consistent with the gene expression profiling from the RNA-seq data.

We also used qRT-PCR to investigate the gene expression patterns of 5 DEGs significantly enriched with phenylpropanoid biosynthesis (Figures 6(c)-6(d)). The expression levels of* SmHCT *(comp50099_c1),* SmF5H *(comp51337_c0),* SmREF1 *(comp24721_c0),* SmCAD1 *(comp48837_c0), and* SmPOD *(comp36960_c0) in BH18 were all higher than that in ZH23, which is also consistent with RNA-seq results.

## 4. Discussion

### 4.1. *S. miltiorrhiza* Transcriptome Study Using RNA-seq

For species without reference genomes, RNA-seq can effectively determine accumulation levels of mRNA that can be associated with gene function, and this may potentially be an underlying cause of phenotypic variation. Previous studies have demonstrated the Sal B concentrations in* S. miltiorrhiza* vary greatly between different genotypes [5, 7, 8]. Thus, the transcriptome profiling of* S. miltiorrhiza *genotypes with different concentrations of bioactive components will promote a relatively accurate understanding of the molecular mechanisms of secondary metabolism in* S. miltiorrhiza*, especially for phenolic acids biosynthesis. However, several previous transcriptome investigations of* S. miltiorrhiza* only used one specific cultivar or a single genotype. In this study, two genotypes with different concentrations of bioactive components were selected to analyze phenolic acids biosynthesis using RNA-seq data.

In RNA-seq, the read lengths and the assembled total lengths are important factors for gene function analysis [20]. In recent years, researchers have undertaken transcriptome studies on* S. miltiorrhiza* using different RNA-seq methods. Yan et al. [14] selected and assembled, using Sanger sequencing, 10,288 expressed sequence tags (ESTs, with sizes ≥ 100 bp) into 4,225 unigenes from the pBluescript II XR cDNA library. Li et al. [15] used the 454 GS FLX platform and obtained 46,722 ESTs with an average read length of 414 bp, which were assembled into 18,235 unigenes. Hua et al. [16] generated 56,774 unigenes (≥200 bp) with an N50 length of 535 bp and a total length of 26.49 Mb by Solexa deep RNA-seq. Using Roche's 454 GS-FLX system, Yang et al. [17] reported a total of 64,139 unigenes with an N50 length of 750 bp and a total size of 26.4 Mb. Gao et al. [18] reported a final total of 20,972 nonredundant genes with a total length of 11.8 Mb. Compared with the previous studies, our Illumina sequencing data showed a longer N50 length (1,577 bp) and a larger coverage of the entire genome with a total length of 47.45 Mb, which provided a more valuable resource for investigating plant development and the biosynthesis of effective components in* S. miltiorrhiza*.

### 4.2. Valuable Clues Regarding the Different Sal B Concentrations in Two* S. miltiorrhiza* Lines

In addition to the phenolic acid-branched pathway, phenylpropanoid metabolism also transforms phenylalanine into a variety of molecules, including lignins and flavonoids such as flavanones, dihydroflavonols, and anthocyanins. Thus, the phenolic acid, lignin, and flavonoid biosynthetic pathways share a common core phenylpropanoid pathway in their early steps.

A total of 25 genes encoding up to 13 enzymes are annotated as being involved in RA biosynthesis in* S. miltiorrhiza*. Some previously reported genes in* S. miltiorrhiza* were generally identified in our RNA-seq data, and most of them showed higher expression in root of ZH23 than that in BH18. A central role gene,* Sm4CL*, in the core phenylpropanoid pathway, was upregulated 1.52-fold in ZH23. In addition, we detected 7 unigenes annotated as “4CL,” excluding comp49431_c1, which was annotated as* Sm4CL2*. Another unigene with relatively low expression levels was annotated as* Sm4CL1* (comp50706_c0), with RPKMs of 5.89 in ZH23 and 2.49 in BH18. This difference in expression is consistent with previous reports that suggest that* Sm4CL2* rather than* Sm4CL1* might be responsible for Sal B biosynthesis in* S. miltiorrhiza* roots [39]. The remaining five 4CL were not previously reported to be present in* S. miltiorrhiza*, and one had the highest level of expression, with RPKMs of 121.72 in BH18 and 146.65 in ZH23; thus, there was no difference between the two lines, and its length was the longest at 3,608 bp. This finding implies that maybe there are more than two functional 4CLs in the* S. miltiorrhiza* transcriptome.

**Figure 7:**
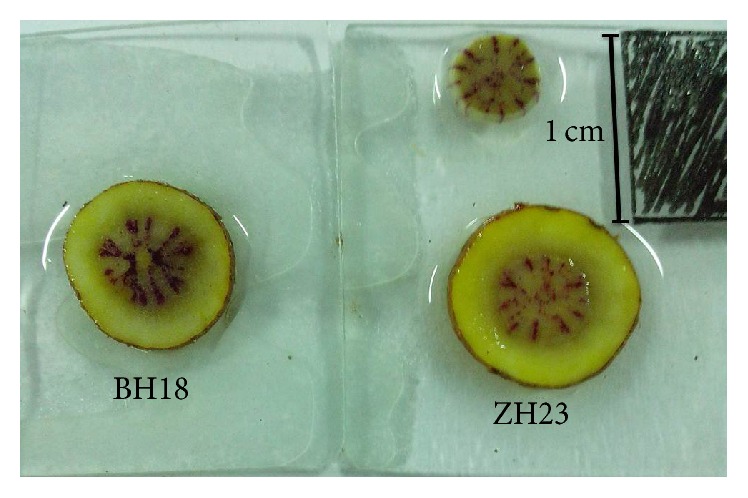
Lignin staining of BH18 and ZH23.

As a result of the downregulation of* Sm4CL2*, and also most of genes in common core phenylpropanoid pathway in BH18, coumarate-CoA co-precursors might have relatively lower expression patterns, thus “reducing income” for the entire downstream pathway. We considered three mainly specific branch pathways: flavonoids, phenolic acids, and lignin. There were no significant DEGs among 28 background unigenes in the flavonoid pathways. To date, the detailed phenolic acid-branched pathway from RA to Sal B has not been characterized. However, the DEGs involved in the lignin pathway indicated it was probably a significantly enriched alternative pathway. Five key enzyme genes in the lignin pathway, for example, HCT, F5H, CAD, REF1, and POX, were upregulated in BH18, which maybe increase lignin biosynthesis to a high level, which can be further shown by histochemical properties and lignin concentrations in roots. We found that a greater number of vascular rays and cells per ray were stained in BH18 (Figure 7), and the Klason lignin concentration in BH18 was higher compared with ZH23 (0.2525 mg/g versus 0.2085 mg/g). Therefore, increasing lignin biosynthesis can be regarded as “increasing expenditure.” We speculated that when compared with BH18, the high Sal B line ZH23 has a higher number of precursors, signifying a decrease in lignin biosynthesis, which allows metabolic flow to be rerouted toward the accumulation of phenolic acids, which ultimately increases the Sal B concentration. It was reported that transgenic* S. miltiorrhiza* plants with downregulated cinnamoyl CoA reductase* (SmCCR)* exhibited dwarfing phenotypes, and both of the S and G lignin monomers were decreased by more than 60%. In contrast, the biosynthesis of phenolic acids, Danshensu, RA, and Sal B, was significantly increased [40]. Zhang et al. [41] also reported that the S lignin concentration decreased approximately 30–40% with ectopic expression of* Arabidopsis Production of Anthocyanin Pigment 1 transcription factor* (AtPAP1) via the cosuppression of* SmCCR* and caffeic acid O-methyltransferase* (SmCOMT)*.

Although these data show that cooverexpression of lignin pathway genes, for example, HCT, CAD, F5H, ERF1, and POD, enhanced the lignin accumulation in BH18, the lower expression of genes in the core common upstream pathway will finally result in lower Sal B and RA contents in BH18 compared with ZH23. This result indicated that expression of genes involved in the core common upstream pathway plays a more important role in downstream second metabolism and also obviously indicated the lignin pathway as potential competing pathway with Sal B biosynthesis.

## Supplementary Material

Table S1: Primers used for gene expression analysis for qRT-PCR.
Table S2: The content of salvianolic acid component in two S. miltiorrhiza lines.
Figure S1: Schematic overview of the phenolic biosynthesis pathway in Salvia miltiorrhiza.
Figure S2: Pathway assignment based on KEGG.
Figure S3: Frequency distribution of BH18 and ZH23 by reads per kilobase per million (RPKM).

## Figures and Tables

**Table 1:** Gene annotation by searching against public databases.

Gene annotation	Number of unigenes	Percentage (%)
Annotated in NR	25,921	46.11
Annotated in NT	9,841	17.5
Annotated in KO	9,162	16.29
Annotated in Swiss-Prot	20,128	35.8
Annotated in PFAM	20,574	36.59
Annotated in GO	22,424	39.88
Annotated in KOG	11,950	21.25
Annotated in all databases	3,375	6
Annotated in at least one database	28,707	51.06
Total unigenes	56,215	

## Abbreviations

TCM: Traditional Chinese medicine
Sal B: Salvianolic acid B
RA: Rosmarinic acid
HPLC: High-performance liquid chromatography
GO: Gene Ontology
COG: Clusters of Orthologous Groups of proteins
KO: KEGG Ortholog database
GRAS: Generally Recognized as Safe
ID: Identifier
KEGG: Kyoto Encyclopedia of Genes and Genomes
KAAS: KEGG Automatic Annotation Server
DEGs: Differentially expressed genes
QRT-PCR: Real-time quantitative reverse transcriptase polymerase chain reaction
RPKM: Reads per kilobase per million
*4CL2*: 4-coumarate:CoA ligase 2
CT: Shikimate O-hydroxycinnamoyl transferase
450: Cytochrome P450-dependent monooxygenase
CAD: Cinnamyl-alcohol dehydrogenase
POD: Peroxidase
CCR: Cinnamoyl CoA reductase
AtPAP1: * Arabidopsis* Production of Anthocyanin Pigment 1 transcription factor
*COMT*: Caffeic acid O-methyltransferase.
