# Supplementary material for: Comparative RNA-Sequence Transcriptome Analysis of Phenolic Acid Metabolism in Salvia miltiorrhiza, a Traditional Chinese Medicine Model Plant
Source: Int J Genomics. 2017 Jan 17;2017:9364594. doi: 10.1155/2017/9364594 (PMC5282420; doi:10.1155/2017/9364594)
Supplement: Supplementary file 1 — Table S1: Primers used for gene expression analysis for qRT-PCR. Table S2: The content of salvianolic acid component in two S. miltiorrhiza lines. Figure S1: Schematic overview of the phenolic biosynthesis pathway in Salvia miltiorrhiza. Figure S2: Pathway assignment based on KEGG. Figure S3: Frequency distribution of BH18 and ZH23 by reads per kilobase per million (RPKM). [file 9364594.f1.doc]

## SUPPORTING INFORMATION


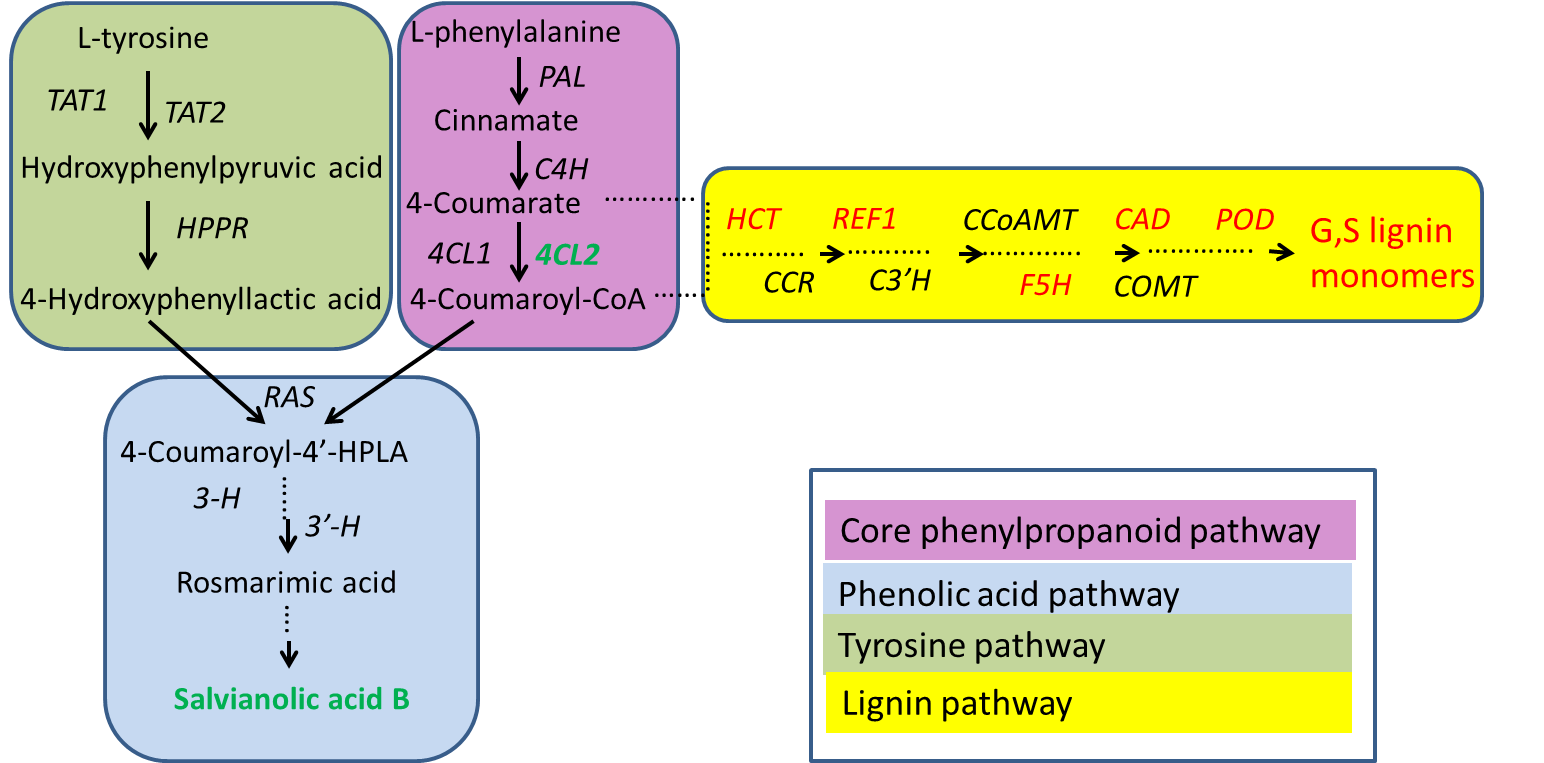


## Figure S1 Schematic overview of the phenolic biosynthesis pathway in *Salvia miltiorrhiza*

Note: Pathway starting with the core phenylpropanoid pathway (pink box) and the tyrosine pathway (green box) and leading to 2 major branch pathways: phenolic acid (blue box) and lignin (yellow box). Up-regulated genes are indicated in red; down-regulated genes are indicated in green; genes with no significant changes in expression are indicated in black. TAT, tyrosine amino transferase; HPPR, hydroxyphenylpyruvate reductase; PAL, phenylalanine ammonia-lyase; C4H, cinnamate 4-hydroxylase; 4CL, 4-coumarate:coenzyme A ligase; RAS, rosmarinic acid synthase; 3’-H, coumaroylquinate (coumaroylshikimate) 3'-monooxygenase; 3-H, coumaroylquinate (coumaroylshikimate) 3-monooxygenase; HCT, hydroxycinnamoyl transferase; CCR, cinnamoyl-CoA reductase; REF1, coniferyl-aldehyde dehydrogenase; CCoAMT, caffeoyl-CoA O-methyltransferase; C3′H, coumarate 3′-hydroxylase; COMT, caffeic acid O-methyltransferase; CAD, cinnamyl-alcohol dehydrogenase; POD, peroxidase. Numbers within gene names (e.g., TAT1 and TAT2) represent different isoforms of gene family members.


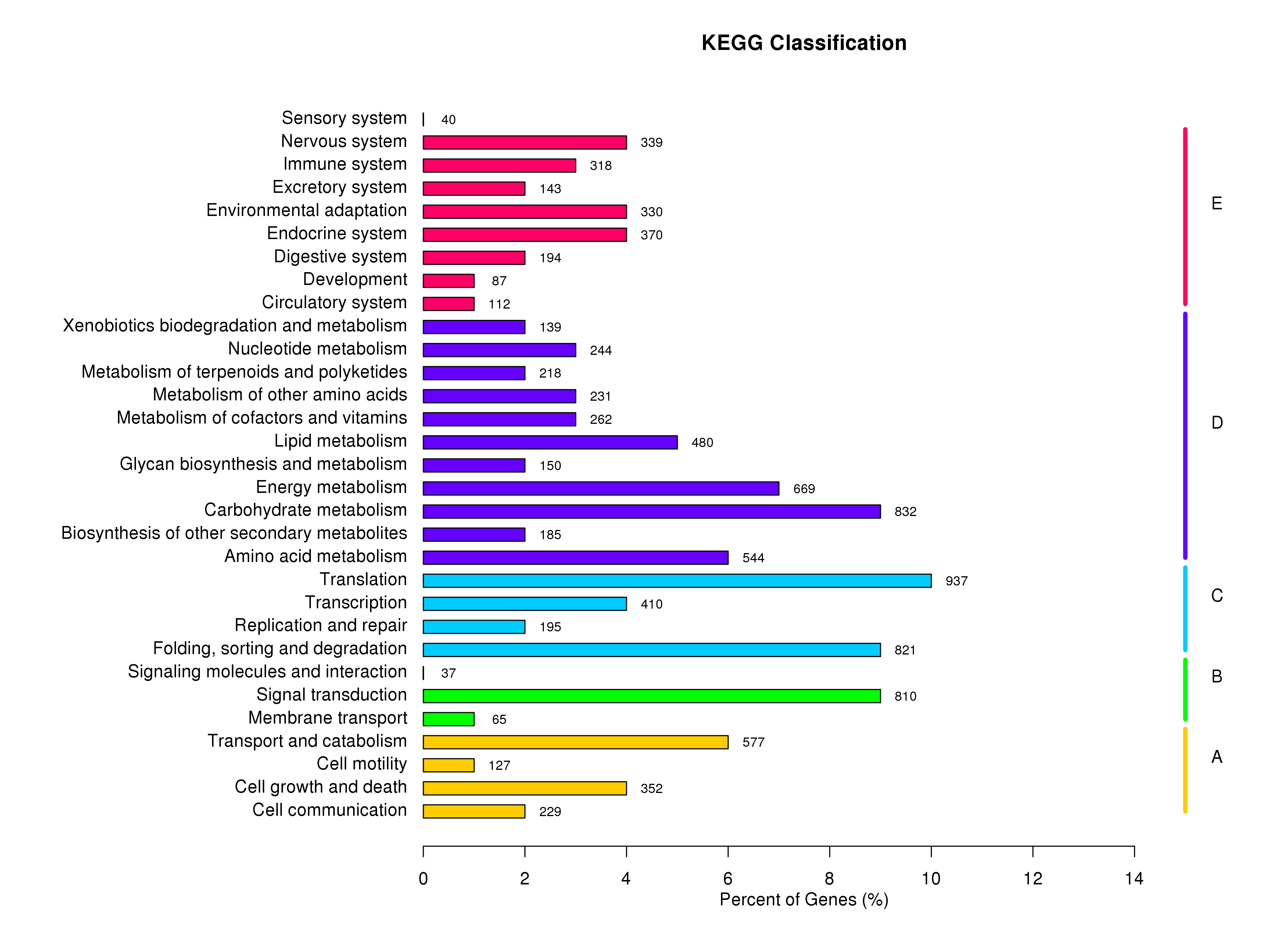


## Figure S2. Pathway assignment based on KEGG.

1. Cellular Processes; (B) Environmental Information Processing; (C) Genetic Information Processing; (D) Metabolism; (E) Organismal Systems


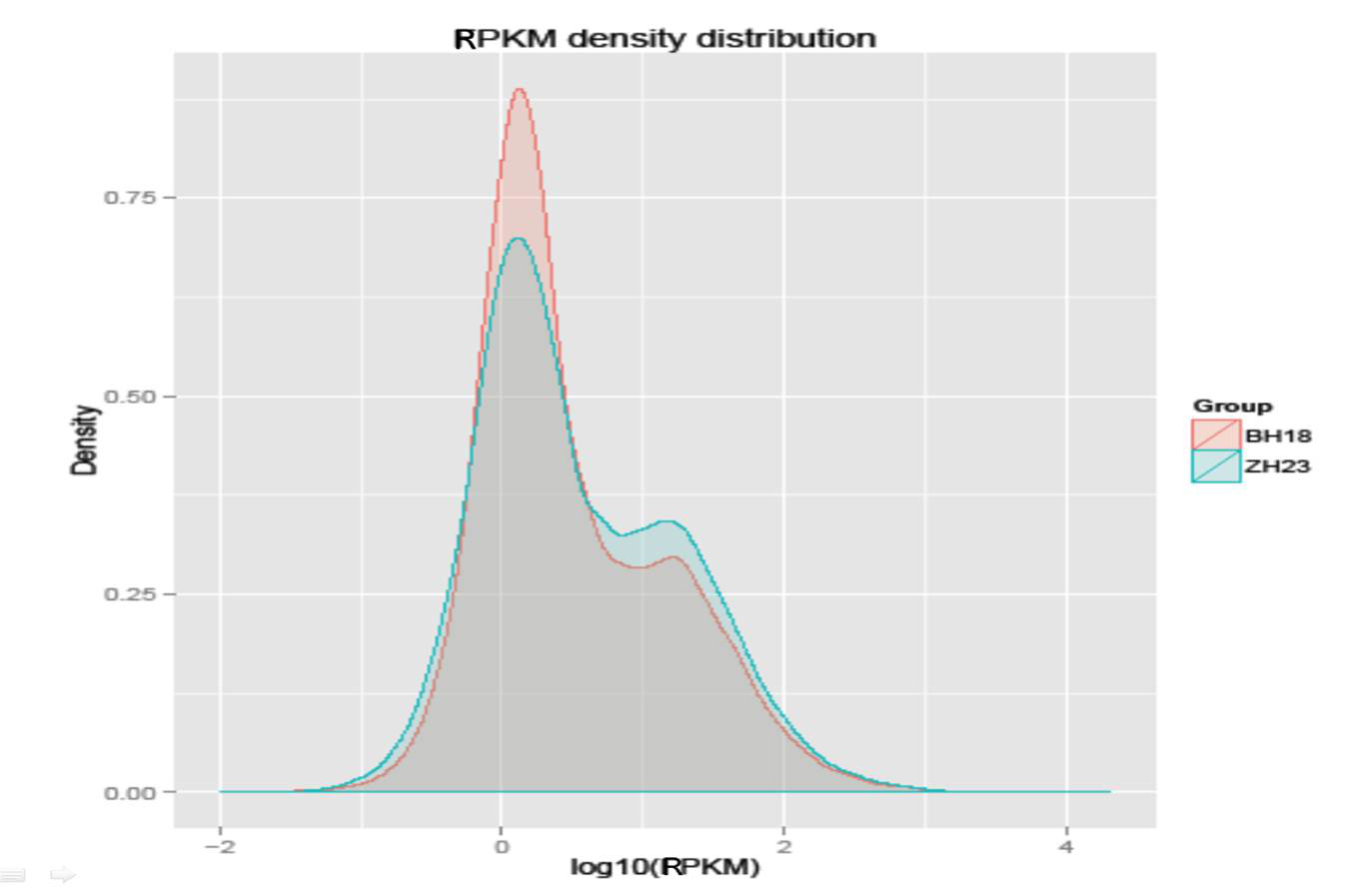


## Figure S3. Frequency distribution of BH18 and ZH23 by reads per kilobase per million (RPKM).
