# Supplementary material for: Comparative RNA-Sequence Transcriptome Analysis of Phenolic Acid Metabolism in Salvia miltiorrhiza, a Traditional Chinese Medicine Model Plant
Source: Int J Genomics. 2017 Jan 17;2017:9364594. doi: 10.1155/2017/9364594 (PMC5282420; doi:10.1155/2017/9364594)
Supplement: Supplementary file 2 [file 9364594.f2.doc]

**Table S1** Primers used for gene expression analysis for qRT-PCR

| Gene | Primer sequence | PCR product (bp) |
| --- | --- | --- |
| *PAL* | F:5'-ACCTACCTCGTCGCCCTATGC-3' | 169 |
|  | R:5'-CCACGCGGATCAAGTCCTTCT-3' |  |
| *C4H* | F:5'-CCAGGAGTCCAAATAACAGAGCC-3' | 186 |
|  | R:5'-GAGCCACCAAGCGTTCACCAA-3' |  |
| *4CL1* | F:5'-ATTCGCATTCGCATTTCTCGG-3' | 162 |
|  | R:5'-GCGGCGTAGTGCTTCACCTTT-3' |  |
| *4CL2* | F:5'-CGCCAAATACGACCTTTCCTC-3' | 133 |
|  | R:5'-GCGGCGTAGTGCTTCACCTTT-3' |  |
| *TAT* | F:5'-TTCAACGGCTACGCTCCAACT-3' | 151 |
|  | R:5'-AAACGGACAATGCTATCTCAAT-3' |  |
| *F5H* | F:5'-AGCAATCTCAGGCTTCGTCAC-3' | 205 |
|  | R:5'-CTCAGCATACTCTGGAGGAATCAC-3' |  |
| *HCT* | F:5'-GTAAAGCCAAAGGCAGAAACG-3' | 181 |
|  | R:5'-AGGGGTAGAAGTCAACCAAAGC-3' |  |
| *CAD* | F:5'-TCAGCACCTCCCCTAACAAGA-3'  R:5'-ATTCCTCCAATCCCACTCCC-3' | 281 |
| *REF1* | F:5'-ACATTGGGATCAAAAGGGTCA-3'  R:5'-GCAAGTCTCCGTTCATCGTCT-3' | 233 |
| *POD1* | F:5'-ATTCCGCCATCCAGAAAGAG-3'  R:5'-GGTCCTCCCAGAATAGAAACAGA-3' | 277 |
| *Ubiquitin* | F:5'-ACCCTCACGGGGAAGACCATC-3' | 207 |
|  | R:5'-ACCACGGAGACGGAGGACAAG-3' |  |

**Table S2 The content of salvianolic acid component in two *S. miltiorrhiza*** lines

|  | Total salvianolic acid content (%) | Rosmarinic acid content (% ) | Salvianolic acid B content (%) |
| --- | --- | --- | --- |
| ZH23 | 11.06%A ± 1.27 | 0.47% ± 0.06 | 8.15%A ± 0.61 |
| BH18 | 6.49%B ± 0.84 | 0.33% ± 0.05 | 3.89%B ± 0.53 |

Note: Significant test followed by LSD, values with different letters are significant at the 5% (lowercase letter) and 1% (capital letter) levels.
